# Altered dopaminergic regulation of the dorsal striatum is able to induce tic-like movements in juvenile rats

**DOI:** 10.1371/journal.pone.0196515

**Published:** 2018-04-26

**Authors:** Ester Nespoli, Francesca Rizzo, Tobias Boeckers, Ulrike Schulze, Bastian Hengerer

**Affiliations:** 1 CNS Department, Boehringer Ingelheim Pharma GmbH& Co. KG, Biberach an der Riss, Germany; 2 Department of Child and Adolescent Psychiatry/Psychotherapy, University of Ulm, Ulm, Germany; 3 Institute of Anatomy and Cell Biology, University of Ulm, Ulm, Germany; Hudson Institute, AUSTRALIA

## Abstract

Motor tics are sudden, repetitive, involuntary movements representing the hallmark behaviors of the neurodevelopmental disease Tourette’s syndrome (TS). The primary cause of TS remains unclear. The initial observation that dopaminergic antagonists alleviate tics led to the development of a dopaminergic theory of TS etiology which is supported by *post mortem* and in vivo studies indicating that non-physiological activation of the striatum could generate tics. The striatum controls movement execution through the balanced activity of dopamine receptor D1 and D2-expressing medium spiny neurons of the direct and indirect pathway, respectively. Different neurotransmitters can activate or repress striatal activity and among them, dopamine plays a major role. In this study we introduced a chronic dopaminergic alteration in juvenile rats, in order to modify the delicate balance between direct and indirect pathway. This manipulation was done in the dorsal striatum, that had been associated with tic-like movements generation in animal models. The results were movements resembling tics, which were categorized and scored according to a newly developed rating scale and were reduced by clonidine and riluzole treatment. Finally, *post mortem* analyses revealed altered RNA expression of dopaminergic receptors D1 and D2, suggesting an imbalanced dopaminergic regulation of medium spiny neuron activity as being causally related to the observed phenotype.

## Introduction

Tics are involuntary movements or vocalizations which change in body localization, frequency, intensity, duration and onset. Chronically active, tics represent the behavioral hallmark of Tourette’s syndrome (TS). TS is a neurodevelopmental disorder that typically manifests at school age, affecting 0.3 to 0.9% of children. TS symptoms last throughout childhood and show a typical waxing and waning course, they decrease after puberty until eventually disappearing in the vast majority of adult patients [1–3]. Motor tics are sudden, brief and meaningless jerks or movements that can be divided into simple tics, when a single muscle or muscle group is involved, or complex tics, when groups of muscles are involved [4].

The underlying mechanism which causes tic development still remains uncertain, but the Cortico Striato Thalamo Cortical (CSTC) circuit controlling movement and behavior appears to have a pivotal role in their progression [5–8]. Within the CSTC circuit, the striatum is commonly recognized as the main regulatory element and numerous studies underlined its role in TS as well [5,9–12].

Medium Spiny Neurons (MSN) are GABAergic projection neurons that make up to 95% of striatal neurons. The remaining 5% comprises various types of interneurons [13] that are thought to maintain a basal state of inhibition within the striatum controlling MSN activation [14,15]. Primates and rodents manifest tic-like movements when MSN are released from interneuronal control [16–18] therefore the regulation of MSN activity could be a crucial factor tic generation.

Multiple neuronal inputs can physiologically modulate MSN activity, but the nigrostriatal dopaminergic system is one of the most prominent. In fact, MSNs express high levels of dopaminergic receptors (DR), mainly the DrD1 and DrD2 subtypes. DRs appear highly segregated on MSNs belonging to the direct or the indirect pathway, respectively [19–23]. These two pathways exert their key role in movement execution by stimulating wanted movements and blocking unwanted ones. Balanced activity of both is required for physiological movement, while perturbed dopaminergic pathways are known to cause movement alterations [24–26].

Furthermore, dopamine (DA) has been the first neurotransmitter to be associated with tics, when administration of DRD2 antagonists such as haloperidol was observed to result in marked tic reduction. Currently, DRD2 antagonists are the only treatments approved by the Food and Drug administration for tics, but are not recommended as first line treatment due to their adverse side-effect profile [27–29].

We hypothesized that a developmental imbalance in the activity of the direct and indirect pathway in the striatum could induce tic-like phenotype in rats. To verify this hypothesis we intervened on the dopaminergic nigrostriatal system of juvenile rats by introducing a lesion of the dopaminergic projections to the dorsal striatum (DS), an area which has previously been associated with tic-like movements generation in animal models [16,30,31]. Chronic stimulation of the DA-deprived area with indirect-pathway agonist quinpirole resulted in altered movements that resembled tics and were reduced by the treatment with clonidine, similarly to what is observed in TS patients [28,29].

## Materials and methods

The present study was conducted in juvenile male Wistar rats (RjHan:WI, Janvier, Le Genest St Isle, France). Fourteen days old rats were housed in groups of 5 together with their mothers under a 12h light and dark cycle in temperature and humidity controlled rooms and with *ad libitum* access to food and water.

The surgical procedure, treatment of animals and their conditions had been approved by the appropriate institutional governmental agency (Regierungspraesidium Tübingen, Germany) and performed in an AAALAC (Association for Assessment and Accreditation of Laboratory Animal Care International)-accredited facility in accordance with the European Convention for Animal Care and Use of Laboratory Animals.

### Experimental design

#### Stereotaxic surgery

Juvenile 21–22 days old rats were anesthetized with 4% isoflurane in N_2_O/O_2_ (70: 30). Rats were adjusted on a stereotaxic frame (David Kopf Instruments, Tujunga, CA, USA) on a flat skull position. Surgical anesthesia was maintained by reducing isoflurane to 1.5–1.2%.

The toxin 6-hydroxydopamine hydrobromide (Sigma-Aldrich, Germany) was dissolved in 0.02% ascorbate solution and injected at a flow rate of 0.5 μL/min into (i) left DS (8μl total injection volume in 2 injection points, at a 2 mg/mL concentration, n = 21), (ii) anterior part of DS (aDS) and (8 μl injection volume, at a 2 mg/mL concentration, n = 15) (iii) central part of DS (cDS) (8 μl injection volume, at a 2 mg/mL concentration, n = 15). Coordinates are calculated from bregma and scaled down from an adult rat brain [32] for the different injection sites (in cm): (i) AP = +0.15 ML = +0.25, DV = -0.52 and AP = -0.02 ML = +0.35, DV = -0.50, (ii) AP = +0.15 ML = +0.25, DV = -0.52, (iii) AP = -0.12 ML = +0.35, DV = -0.52. Sham rats were injected in the left dorsal striatum (i) with ascorbate solution (n = 15). For all animals, the needle was left in place for 3 additional minutes after injection and then retracted. Analgesia was provided with Meloxicam (1mg/kg SC in 2mg/ml injection volume, Boehringer Ingelheim Vetmedica GmbH, Germany) 20 minutes before the surgical procedure and at the end. After recovering from surgery, animals were separated from their mothers and housed in groups of 5 siblings to prevent isolation.

### Dopamine agonist priming and phenotype score

Thirteen days after surgery chronic treatment with a solution of quinpirole (0.5 mg/kg in saline, IP) for a total of 7 times over 15 days was initiated, as to induce and stabilize the motor phenotype. On the last day of treatment, rats were individually observed for 1min every 30mins and their phenotype was scored according to our newly developed scale.

### Drugs preparation and testing

Quinpirole hydrochloride, clonidine and riluzole were obtained from Sigma-Aldrich Chemie GmbH, Germany. Haloperidol was purchased from Janssen-Cilag GmbH (Germany) and was diluted in saline at the desired dose (0.1 mg/kg).

Quinpirole was freshly dissolved in saline shortly before administration (IP, 0.5mg kg).

To investigate the effect of clonidine and riluzole on quinpirole-induced motor phenotype, drugs were injected together or after quinpirole administration in a way that the maximal effect of the injected drug would coincide with the phase of constant tic-like phenotype, that took place 60–120 min after quinpirole administration. Riluzole (2-amino-6-trifluoromethoxy benzothiazole) was dissolved in 1% tween-20 solution and left overnight under magnetic stirring. On the test day, riluzole was injected (6 mg/kg IP) 60 minutes after quinpirole administration, tic-like phenotype score of quinpirole treated aDS, cDS and DS lesioned juvenile rats was used as control. Clonidine was freshly dissolved in saline and injected together with quinpirole (IP, 0.5 and 0.05 mg/kg).

### Haloperidol-induced catalepsy

Haloperidol-induced cataleptic immobility is a well-established measure of extrapyramidal side effects typically associated with antipsychotic treatment [33].

After 45 min from quinpirole administration, rats (n = 8 each group) were injected with saline as a negative control, haloperidol for positive control (0.1, 0.5 or 1 mg/kg IP) or riluzole (6 mg/kg IP). After 15 more minutes they were placed with their forepaws on a horizontal bar in bipedal position for 1min. In case the rat left the bar within the observation time, it was replaced on it for a maximum of four times. The total time of immobility on the bar was recorded as time of catalepsy.

### Food intake measurement

During phenotype score, quinpirole treated DS lesioned juvenile rats were provided with a previously weighed amount of food in the observation cage. After 3hrs the remaining food was carefully removed and weighed. The difference between the initial and the final amount of food was considered as food intake measure. The same experiment at the same circumstances was repeated with DS lesioned juvenile rats administered with saline.

### Ultrasound vocalization quantification

One hour after saline (n = 7) or quinpirole (n = 8) administration, rats were individually placed with their cage in a sound-proof chamber. Spontaneous ultrasonic vocalizations (USV) produced for 5mins were recorded through a condenser microphone (CM16; Avisoft Bioacoustic, Germany), mounted 20 cm above the rat’s cage center, by Avisoft recorder Software (UltraSound Gate 116 USB, version 3.2 Avisoft Bioacoustics, Germany). The system was sensitive to frequencies of 10–180 kHz, selected sampling frequency was 300 kHz and 16 bit was the format chosen. The obtained WAV (.wav) file recordings were analyzed through SAS Lab pro Software (version 4.5, Avisoft Bioacoustics) and the average number of total calls, amplitude (in kHz), peak frequency (in kHz) and duration (in sec). Calls were further categorized into 22 kHz, 50 kHz monosyllabic and 50 kHz complex calls, representing 50 kHz calls constituted of more than one syllable, and their average percentage was calculated over the total calls.

### *Post mortem* analyses

#### *RNAscope*^®^ *in situ* hybridization

A total of 6 rats ware used for DRD1 and DRD2 RNA analysis with *in situ* hybridization. Rats were sacrificed at PND25 (n = 3) shortly after stereotaxic surgery, or at PND50 (n = 3), after priming and phenotype verification. For this group, a solution of 0.5 mg/kg of quinpirole had been administered 90mins prior to sacrifice. Rats were deeply sedated with isoflurane and sacrificed by decapitation, the brain was readily extracted, snap frozen in liquid nitrogen and stored at -80°C. Samples had been moved at -20°C on the night before sectioning and multiple 12μm cryosections from aDS and cDS were taken, allowing the same brains to be independently stained for DRD1 and DRD2 RNA.

For each timepoint, 3 different sections (in correspondence with aDS and cDS injection sites, and an intermediate section), were used for quantification, for a total of 9 sections quantified for DRD1 and DRD2. One aDS section was later excluded from quantification because of its damage during the procedure.

*In situ* hybridization was performed using the RNAscope^®^ 2.5 HD Red Chromogenic Reagent Kit (Advanced Cell Diagnostics, Hayward, CA ACD#322350), that allowed detection of target RNA at single cell level. Probes for rat DRD1 (RNAscope^®^ Probe- Rn-Drd1a ACD# 317031) and DRD2 (RNAscope^®^ Probe- Rn-DRD2 ACD# 315641) were designed and provided by the manufacturer and the experimental procedure followed the manufacturer’s instructions for single plex assay. Briefly, frozen cryosections were fixed by immersion in 4% PFA at 4°C for 15 mins. The tissue was dehydrated in EtOH (50%, 70% and 100%), permeabilized with RNAscope^®^ Hydrogen Peroxide for 10 minutes at RT (ACD# 322335) and pretreated RNAscope^®^ Protease IV (ACD# 322336) for 30 minutes at room temperature.

Target probes for DRD1 and DRD2 were independently hybridized for 2 h at 40°C and the signal was later amplified through 6 consecutive steps. A chromogenic enzymatic reaction produced a red signal in correspondence with the hybridized probe, while hematoxylin was used for nuclear counterstaining. Images were acquired using ZEISS Axio Scan.Z1 and analyzed with HALO image analysis platform (Indica labs). For quantitative evaluation, the areas of interest were manually selected: on the lesioned side the analyzed area corresponded with the lesion (the area was chosen through comparison with tyrosine hydroxylase immunostaining) and an analogous area was selected on the control side. HALO software segregates cells into negative (only hematoxylin staining) and positive cells for hematoxylin and DrD1 or DrD2 probes based on the nuclear optical density (minimal OD 0,338). Positive cells were reported as percentage of the total cells.

#### Immunohistochemistry

The presence of tyrosine hydroxylase (TH) the rate-limiting enzyme for DA synthesis was analyzed by immunohistochemistry to detect the lesioned area in the DS of PND56 days old rats. After phenotype verification, at PND50 deeply anesthetized rats were transcardially perfused with saline and by 4% ice-cold paraformaldehyde. Brains were rapidly extracted, post-fixed in the same solution for 3 hours and then stored in 30% sucrose at 4°C. After the samples had sunk in the sucrose solution they were frozen with dry ice and stored at -80°C until use. Coronal sections of 12μm thick of aDS and cDS regions were sectioned using a cryostat at −20°C. After fixation for 20mins in 4% paraformaldehyde slides were blocked for 2h with a solution of 1% bovine serum albumin, 0.045% fish gelatin and 0.1% saponine. Samples were incubated overnight with mouse monoclonal anti-TH primary antibody (1:400, Millipore Cat# MAB5280, RRID: AB_2201526), of which the specificity is routinely assessed by the manufacturer. After washing, the primary antibody was detected with Alexa Fluor^®^ 488 (Thermo Scientific, Molecular Probes Cat# A-11017, RRID: AB_143160). Fluorescent brain images were taken through Typhoon^™^ 9400 Variable Mode Imager (GE Healthcare).

### Statistical analyses

All statistical analyses were performed using GraphPad Prism 7 (GraphPad Software, San Diego, CA, USA). Data are expressed as mean ± SEM and P values equal to or less than 0.05 were considered to be statistically significant. All time-effect data were analyzed for statistical significance using two-way analysis of variance (ANOVA) followed by Sidak´s test for multiple comparison. Phenotype components in aDS cDS and DS was analyzed through one-way ANOVA and Dunn´s multiple comparison tests. DrD1 and DrD2 RNA expression and cell density variations between PND25 and PND50 were analyzed with repeated measures two-way ANOVA corrected by Sidak´s multiple comparison´s test. Total cell density in lesioned versus control side and food intake of quinpirole or saline treatment rats were compared with paired Student’s t test. Number, duration, frequency and amplitude of ultrasonic vocalizations as well as time of catalepsy were analyzed with one-way ANOVA and Dunn’s test for multiple comparisons. Types of USV are expressed in percentage of the total number and are analyzed with two-way ANOVA followed by Tukey´s test for multiple comparisons.

## Results

### Quinpirole chronic treatment of juvenile rats lesioned with 6-OHDA in dorsal striatum induced tic-like movements

**Fig 1:**
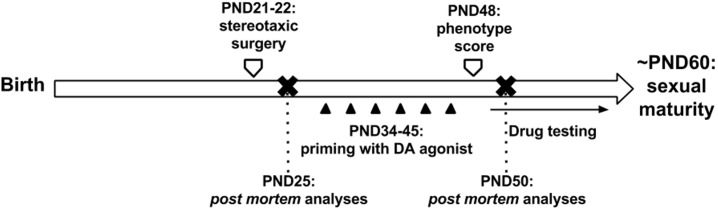
Experimental design. The degeneration of nigrostriatal dopaminergic projections on the dorsal striatum was achieved through stereotaxic injection of the toxin 6-hydroxydopamine (6-OHDA) at postnatal day 21 (PND21). After recovery, rats were chronically administered with the DA agonist quinpirole that induced a tic-like motor phenotype that was quantified on PND48 and used for drug testing. The entire experimental procedure was concluded before puberty (~PND60-70).

Chronic DRD2/ DRD3 agonist quinpirole administration to juvenile rats lesioned with 6-OHDA in the dorsal striatum (DS) (Fig 1) gave raise to abnormal movements that were carefully observed and summarized in Table 1.

**Table 1:** Tic-like movements observed in 6-OHDA lesioned juvenile rats chronically administered with quinpirole. Simple tic-like movements involve the mouth, neck and limb contralateral to the lesion. Complex tic-like movements are movements of the mouth, neck and limb that occur simultaneously in a repeated and fixed pattern.

	**Movements resembling TICS**	
	Sudden jerks or movements that recur in bouts and involve a single muscle or muscle group **(SIMPLE TICS**)	Sudden jerks or movements involving multiple body parts recurring in a clear and repeated pattern **(COMPLEX TICS)**
Mouth	biting sniffing tongue protrusion licking	biting (and/or) tongue protrusion + side to side head twitching + side-to-side movements of the contralateral front paw under the chin (and/or) opening and closing movement of the contralateral paw
Neck	side to side head twitching	
Contralateral limb	side-to-side movements of the contralateral front paw under the chin opening and closing movement of the contralateral paw	
	**Other movements**	
	Contralateral rotations Dystonic postures	

Abnormal movements followed quinpirole pharmacokinetics and started quickly after quinpirole administration, peaking in intensity between 30- 150mins and slowly reducing to an end around 4hrs after administration.

Movements appeared sudden, fast and occurred in bouts during observation time but their overall score remained almost constant for more than 2hrs after quinpirole administration and before progressively declining. Abnormal movements did not impair the physiological behavior of the animal that was normally able to eat and drink (S1 Fig), walk (except fora very mild contralateral rotation behavior), and engage in social behavior if reunited with its siblings.

To quantify tic like movements we needed to develop a rating scale as no rating system for tic-like movements in rodent models existed. For this purpose, we adapted the Yale Global Tic Severity Scale (YGTTS) used for TS patients to our rodent model. The YGTTS takes into account five separate aspects of motor tics: number, frequency, intensity, complexity, and interference with normal activities. The same parameters were taken into account for tic quantification in our animal model, except for frequency and intensity that in our case form a single parameter because of the inducible nature of the model.

Three body parts were identified as candidates to show tic-like movements: mouth, neck and the paw contralateral to the lesion. The frequency/ intensity represents the duration of tic bouts over pauses, when the animal continues its natural behavior (walking, eating, grooming etc.). This parameter is calculated independently for each of the body parts involved as follows: 0 = tic-like movements are absent, 1 = mild (present in less than 40% of time, long pauses interpolate few tic bouts), 2 = frequent (present in 40–70% of time, tic bouts and pauses equally alternate) 3 = continuous (almost present at all times with only short spontaneous interruptions), 4 = compulsive (always present with no interruptions).

One body part can show tic-like movements alone or at the same time with other body parts but independently, similarly to simple tics in patients which involve single muscles or muscle groups. Furthermore, we observed repetitive movements of more than one body part together which were repeated in a clear pattern. We recorded these as complex tics.

The overall degree of complexity of tic-like movements was measured as follows: 0 = only simple tics present, 1 = complex tics appear but represent less than 30% of the total time spent performing tics, 2 = complex tics represent less than 60% of the total time spent performing tics, 3 = continuous presence of complex tics with rare and short spontaneous interruptions (60–90% of the total tics), 4 = compulsive complex tics cannot be interrupted by the animal or by external stimuli.

Finally, the interference of tics with normal rat´s behavior is taken into account and considered as value of impairment of the phenotype. This parameter was rated as follows: 0 = the animal is able to freely alternate tic behavior with his natural behavior, 1 = the animal is exclusively engaged in tic-like activity that lasts for less than half of the observation time (less than 30sec) with no interruption, and/or it shows strong axial torsion for less than 20sec in total, and/or shows continued rotational behavior for less than 15 consecutive seconds. 2 = points the animal is exclusively engaged in tic activity for more than half of the observation time with no interruption and/or it shows strong axial torsion for more than 20sec in total, and/or shows continued rotational behavior for more than 15 consecutive seconds.

The total tic-like movement score is calculated as a sum of the number of body parts (0 to 3 points), the average of the frequency/ intensity of the different body parts (0 to 4 points), complexity of tics (0 to 4 points) and impairment (0 to 2 points) as shown in Table 2. Borderline scores such as 1.5, 2.5 and 3.5 are allowed for frequency/intensity and complexity parameters to improve the sensitivity of the scale.

**Table 2:** Tics rating parameters and related scores. The rating parameters used are translated from the YGTSS used for patients, and include the number of body parts, frequency of the tic-like movement, complexity and impairment of the normal rats’ behavior.

Number of body parts 0–3 points	Frequency 0–4 points	Complexity 0–4 points	Impairment 0–2 points
• Limb (+1)	Calculated as average of: • Limb frequency/ intensity: 0 = none 1 = mild 2 = frequent 3 = continuous 4 = compulsive	0 = none 1 = mild 2 = moderate 3 = marked 4 = compulsive	0 = none 1 = mild 2 = strong
• Neck (+1)	• Neck frequency/ intensity: 0 = none 1 = mild 2 = frequent 3 = continuous 4 = compulsive		
• Mouth (+1)	• Mouth frequency/ intensity: 0 = none 1 = mild 2 = frequent 3 = continuous 4 = compulsive		

**Fig 2:**
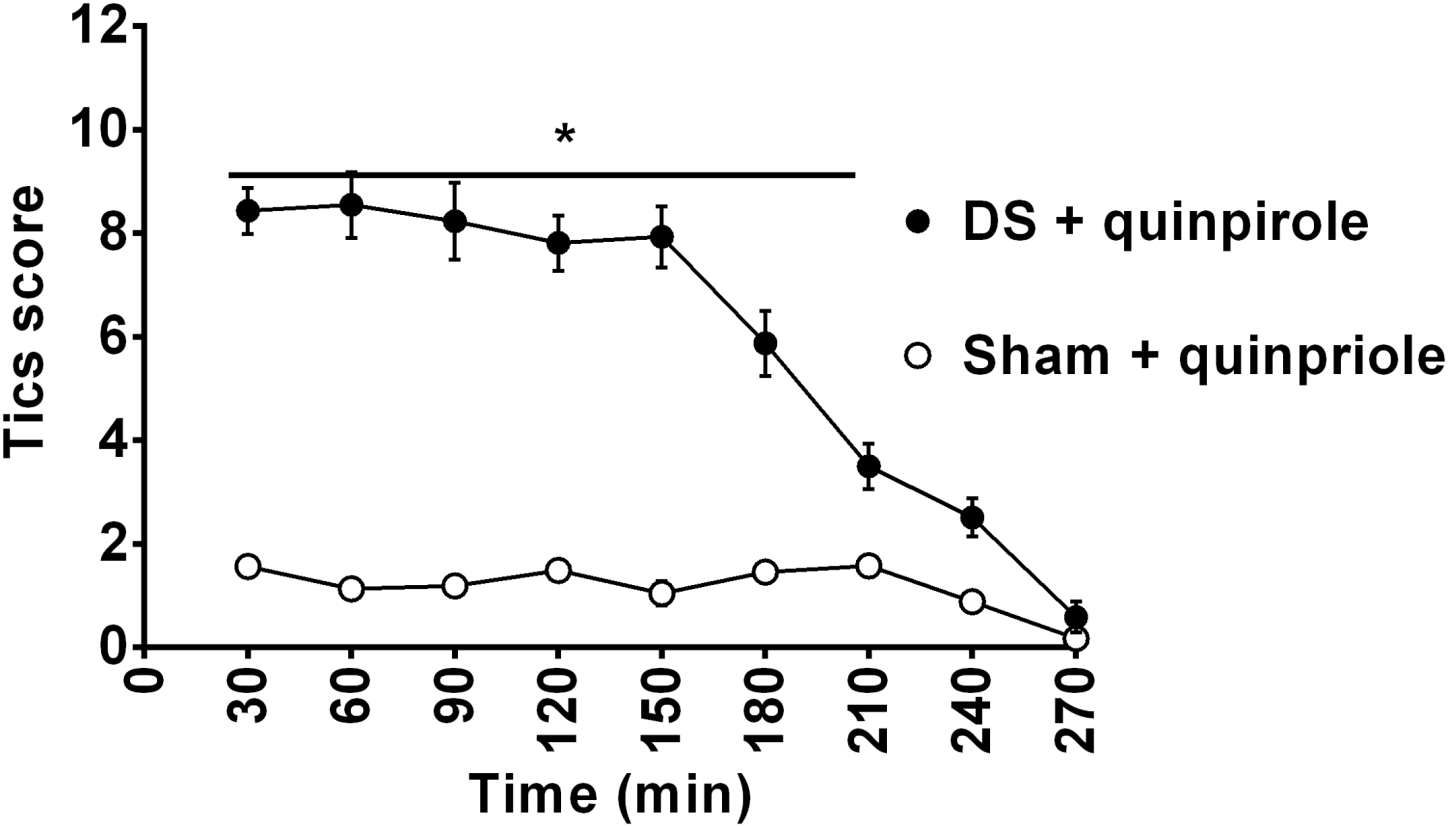
Time course of tic-like movements score of quinpirole-treated juvenile rats lesioned in the dorsal striatum (DS) and unlesioned rats. On observation day, tic-like movements score is taken every 30 min after quinpirole administration (0.5 mg/kg) and is calculated as the total number of body parts + average frequency + complexity + impairment scores. A significant difference between tic-like score in quinpirole treated lesioned and sham rats is indicated as *p< 0.05.

The rating scale was tested to quantify the phenotype associated with quinpirole treatment in juvenile rats with lesion of dopaminergic nigrostriatal projections on the DS. Results show significantly different tic-like movements score between sham and DS rats (F_(1,28)_ = 204.6, p<0.0001) and between time points (F_(9,252)_ = 65.40, p<0.0001), with a significant interaction between groups and time (F_(9,252)_ = 40.03, p<0.0001). (Fig 2).

During the observation period, juvenile rats were engaged in simple and complex tic-like movements of the contralateral limb, neck and mouth that did not interfere with food consumption (S1 Fig). In contrast to mouth and limb movements, neck movements did not appear as simple tic-like movements; rather they only appeared in concert with limb and mouth movements to form complex tic-like movements.

**Fig 3:**
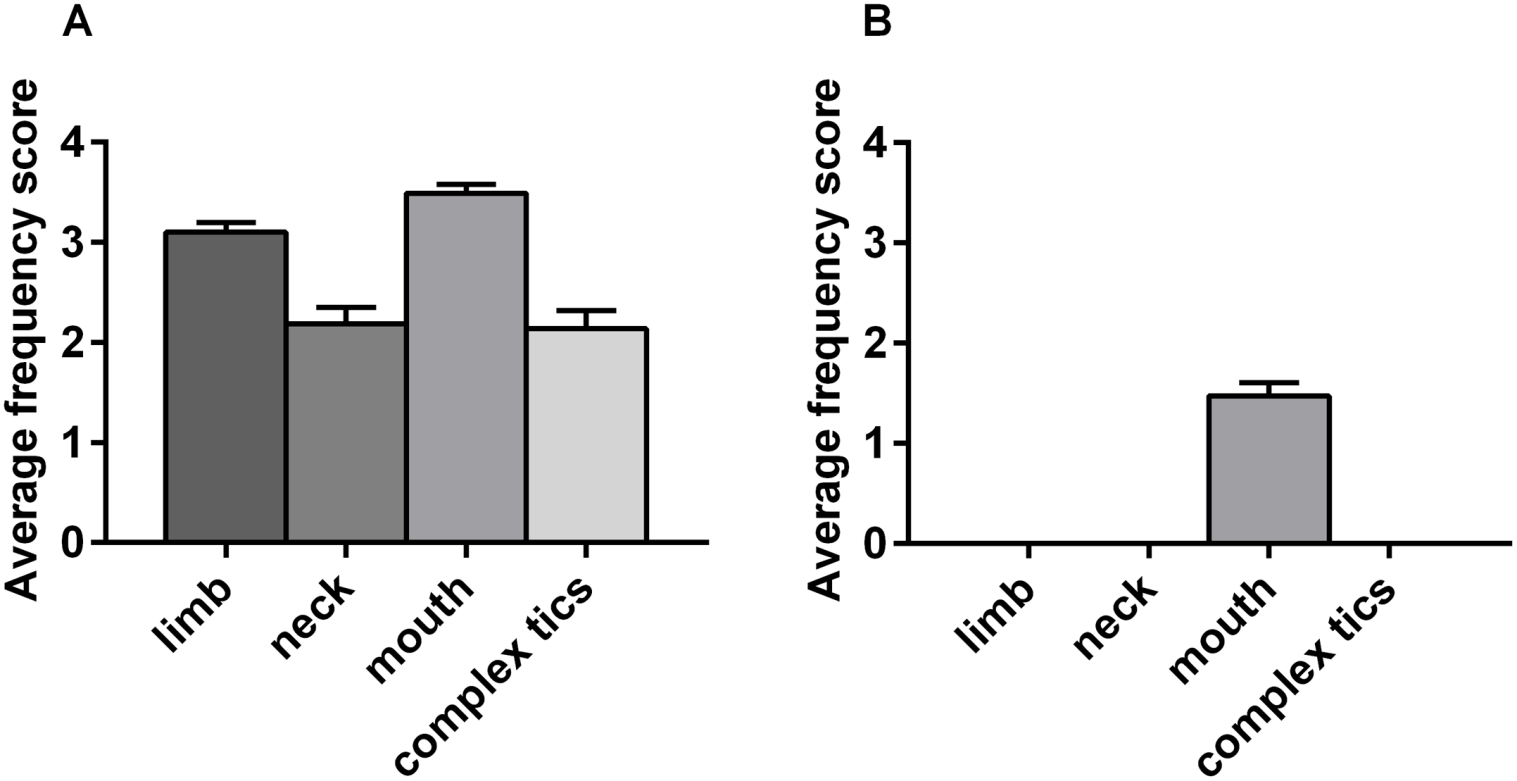
Average frequency scores of the body parts involved in tic-like movements. Average frequency scores of limb, neck, mouth movements and complex tic-like movements during the phase of maximal abnormal motor manifestation (60-120min after quinpirole administration) on dorsal striatum-lesioned rats (A) or sham-lesioned rats (B).

Quinpirole-induced repetitive chewing and biting movements in sham-lesioned group suggesting that DRD2 activation alone can be sufficient for oral movements induction, in agreement with previous reports [34–36] (Fig 3), but no difference in the type, number, duration, frequency and amplitude of USV was registered between DS lesioned and sham rats treated with quinpirole (S2 Fig).

### Anterior and central dorsal striatum differentially contribute to the motor phenotype

The phenotype described above was achieved by two separate injections of 6-OHDA: one in the anterior part (aDS) and the second in the central part (cDS) of the DS (S4 and S5 Figs). As the striatum reflects the topographic organization of the movement circuit [37], we decided to investigate the two injection sites independently and observe their contribution to the motor phenotype.

**Fig 4:**
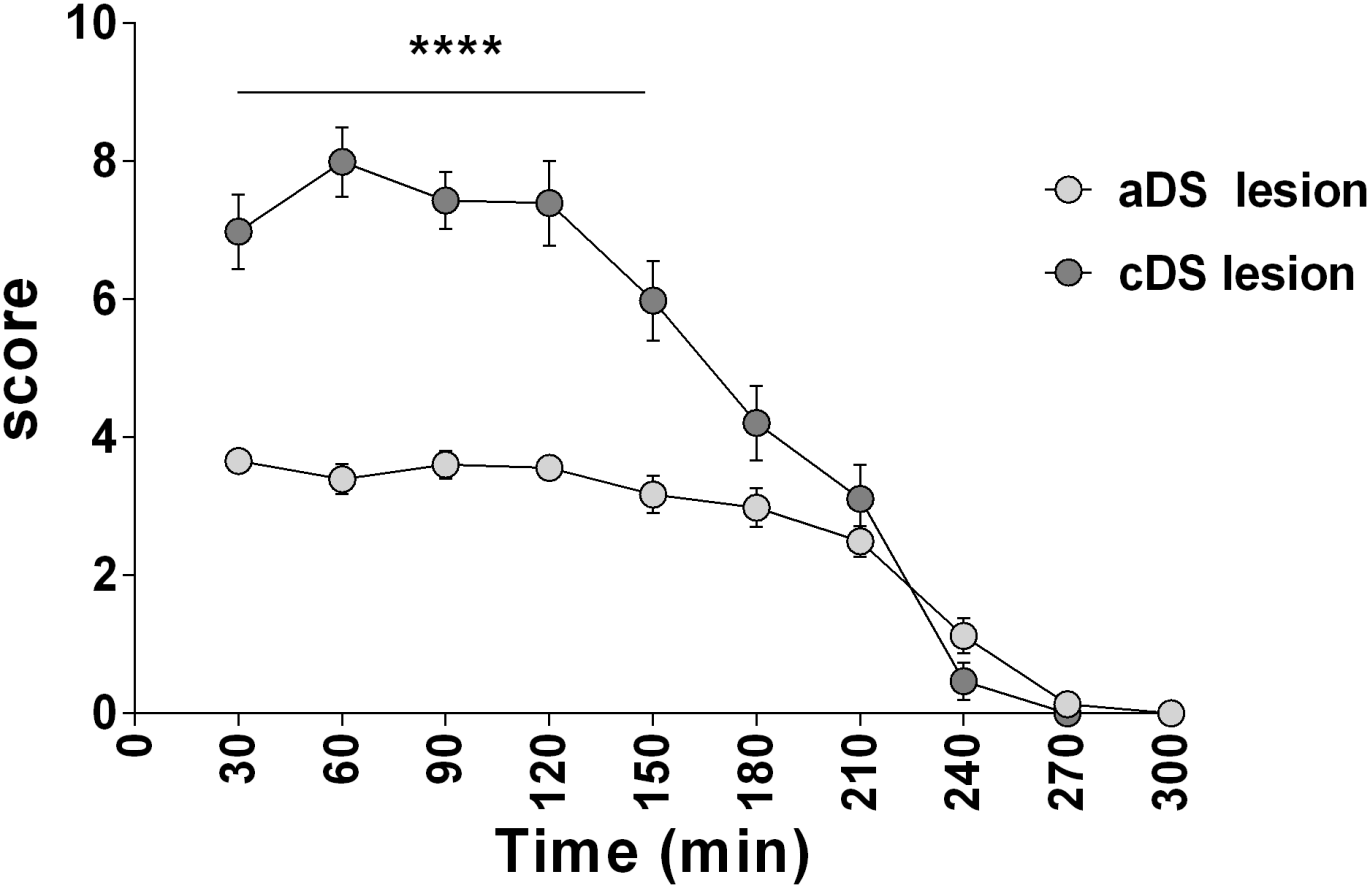
Time course of tic-like movements score in aDS and cDS. Tic-like movements score is taken every 30 min after quinpirole administration and is calculated as the total of number of body parts + average frequency + complexity + impairment scores. Significant difference is indicated as ****p<0.001.

Total tic-like movements score in aDS was significantly milder than in cDS lesioned juvenile rats (F_(9,280)_ = 281.0, p<0.0001) and between time points (F_(1,280)_ = 133.6, p<0.0001), with a significant interaction between groups and time (F_(9,280)_ = 10.12, p<0.0001) (Fig 4)

**Fig 5:**
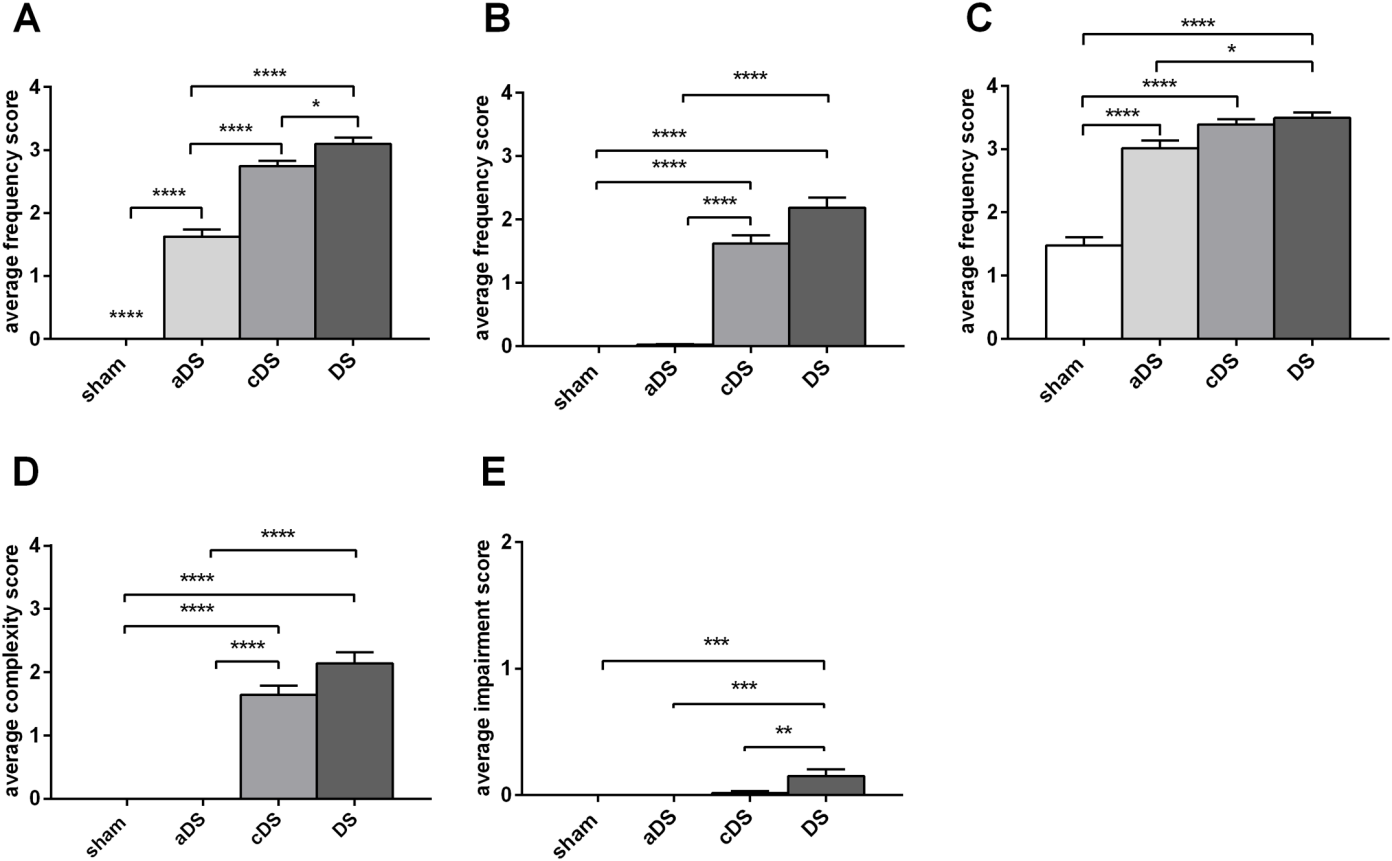
Phenotype components in DS, aDS and cDS lesioned juvenile rats. The average frequency scores, the average complexity score and the average impairment score are calculated during the phase of maximal phenotype score (60- 120mins after quinpirole administration) for the different body parts involved in tic-like movements: limb (A), neck (B), mouth (C), complex tic-like movements (D) and impairment score (E). Significant difference is indicated as *p<0.05 **p<0.01 ****p<0.001.

Juvenile rats lesioned in aDS showed a mild phenotype consisting of simple tic-like movements of the mouth and the limb (Fig 5).

CDS lesioned juvenile rats showed an intense and complex phenotype similar to DS lesioned juvenile rats but with a significantly shorter duration (time points 150 and 180 min men diff = 2, p<0.005 for 150min and mean diff = 1.7, p<0.01. Figs 2 and 4). The phenotype of all considered body parts of cDS lesioned juvenile rats was intermediate to this of aDS and DS lesioned juvenile rats, and in all three groups the tic-like phenotype did not impact on the normal rats’ behavior (Fig 5).

### DRD1 but not DRD2 RNA expression is reduced in pre-pubertal rats lesioned in the dorsal striatum

**Fig 6:**
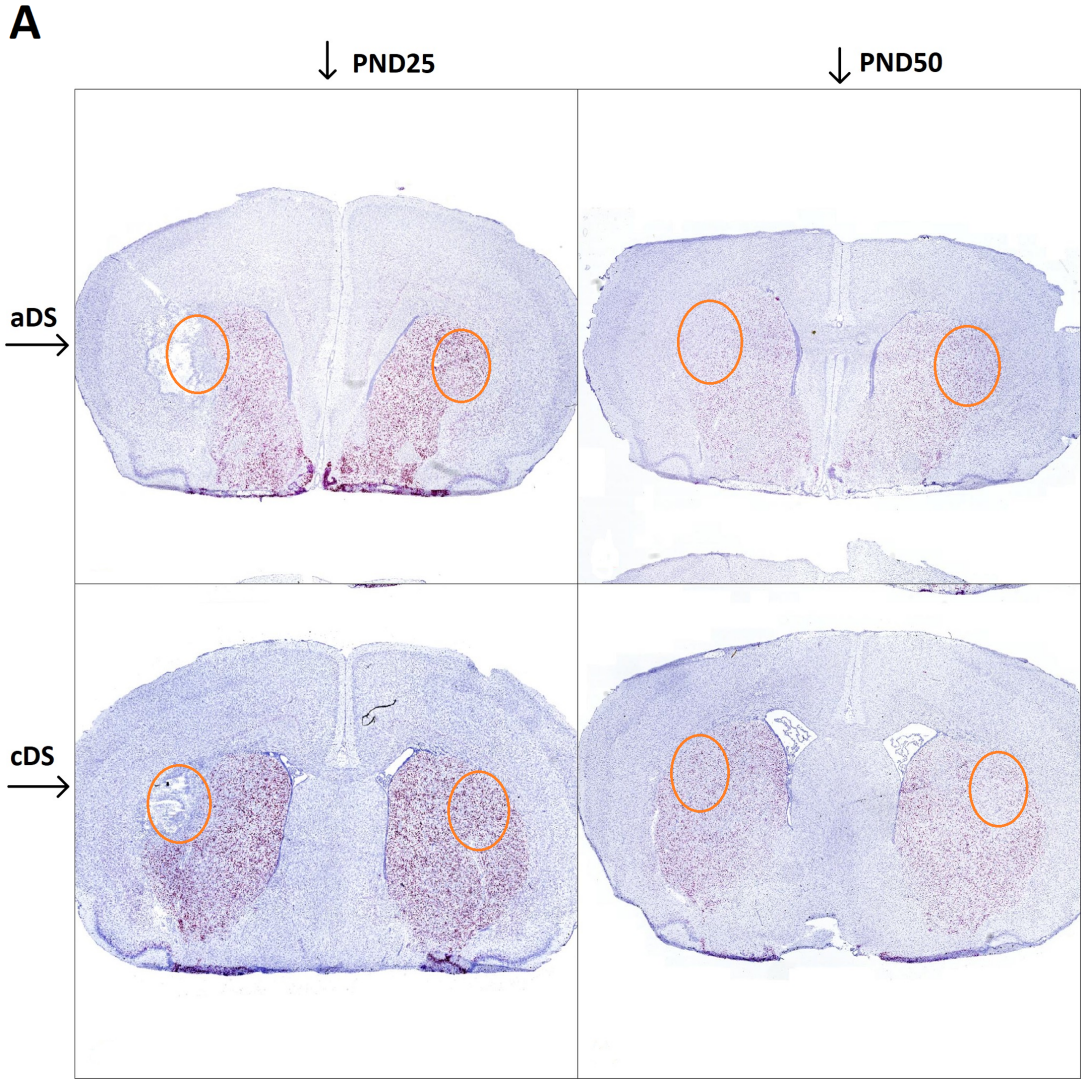
*In situ* hybridization reveals DrD1 RNA expression in aDS and cDS of 6-OHDA lesioned rats, evaluated at PND25 and PND50. In all panels, red dots result from a chromogenic reaction indicating the presence of the target RNA, while blue dots represent the nuclear marker; aDS PND25 (top left), cDS PND25 (bottom left), aDS PND50 (top right), cDS PND50 (bottom right). The orange circle approximately indicates the area taken into account for quantitative analysis.

**Fig 7:**
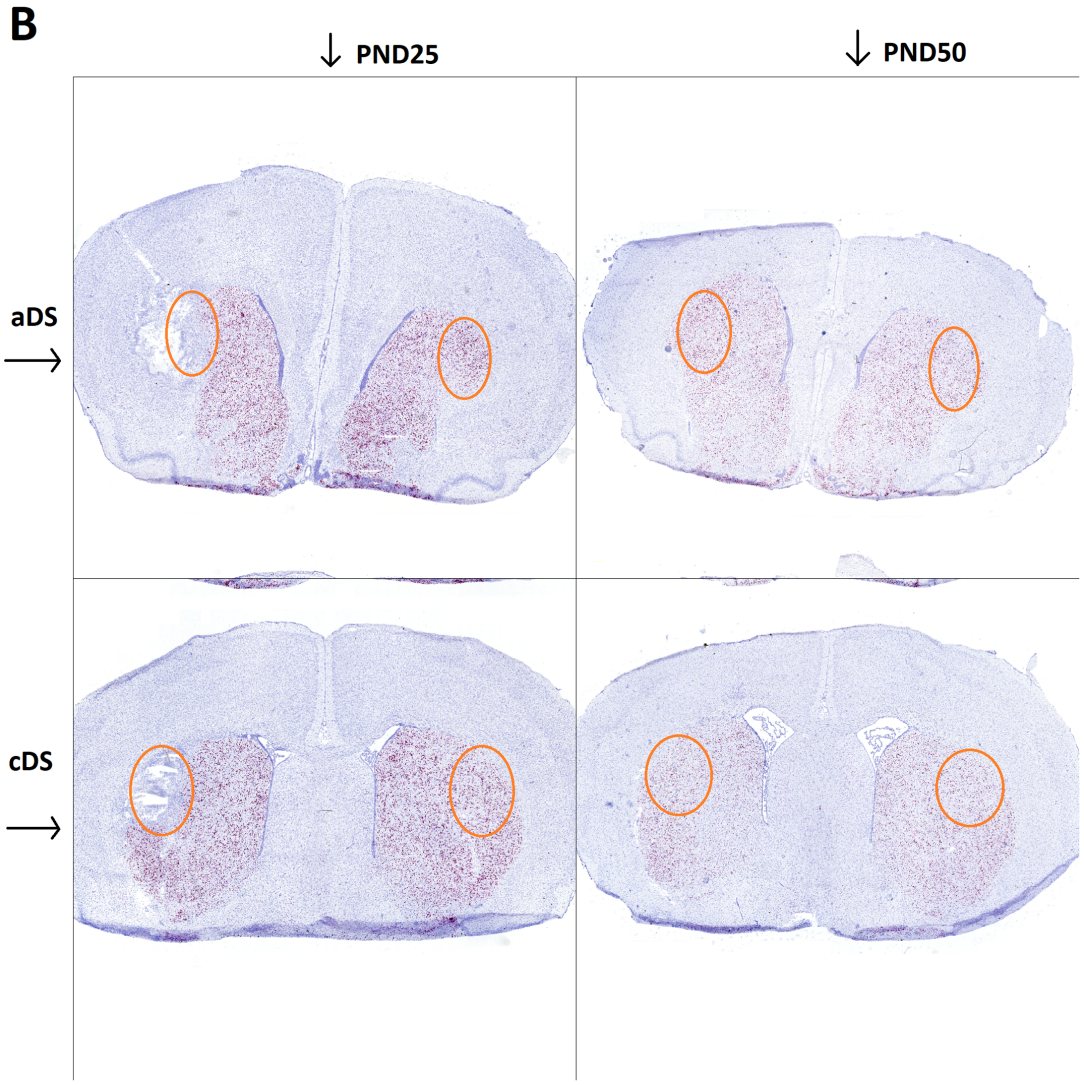
*In situ* hybridization reveals DrD2 RNA expression in aDS and cDS of 6-OHDA lesioned rats, evaluated at PND25 and PND50. In all panels, red dots result from a chromogenic reaction indicating the presence of the target RNA, while blue dots represent the nuclear marker; aDS PND25 (top left), cDS PND25 (bottom left), aDS PND50 (top right), cDS PND50 (bottom right). The orange circle approximately indicates the area taken into account for quantitative analysis.

We hypothesized that motor tic-like phenotype was associated with a dopaminergic imbalance which influences activation and functioning of the direct and indirect pathway in the developing striatum. For this reason, we investigated the level of DRD1 or DRD2 RNA expression in juvenile rats at PND25 shortly after 6-OHDA lesion in DS at PND21, and at PND50, in pre-pubertal rats with a stable tic-like phenotype. We addressed this question via high resolution RNAscope^®^ technology, which allowed for marking of postsynaptic cell nuclei with hematoxylin (blue dots), and to visualize and quantify cells expressing the RNA of interest (red dots and positive nuclear staining) at a single cell level (Figs 6 and 7).

**Fig 8:**
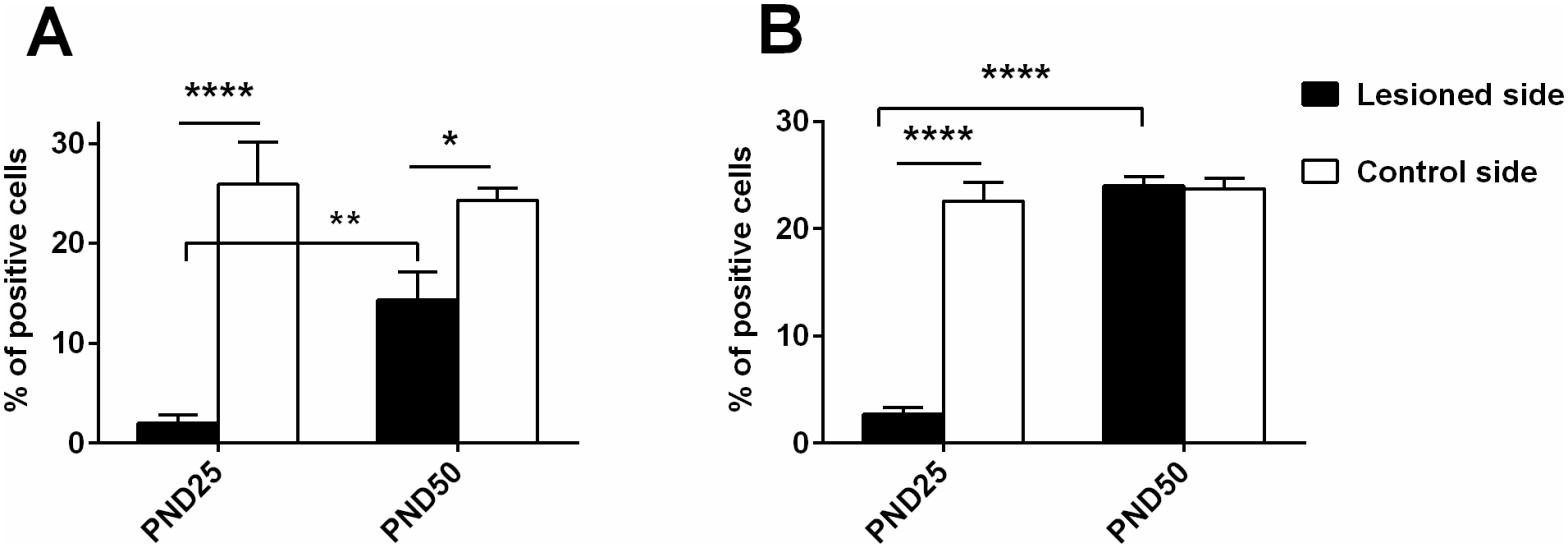
Percentage of DRD1 and DRD2 positive cells in DS. The number of positive cells for DRD1 RNA and nuclear staining (A) or DRD2 RNA and nuclear staining (B) in the lesioned and in the corresponding area on the control side of PND25 and PND50 rats is reported in percentages of the total number of nuclear staining-positive cells. Significant total difference is indicated as ****p<0.001, **p<0.01, *p<0.05.

**Fig 9:**
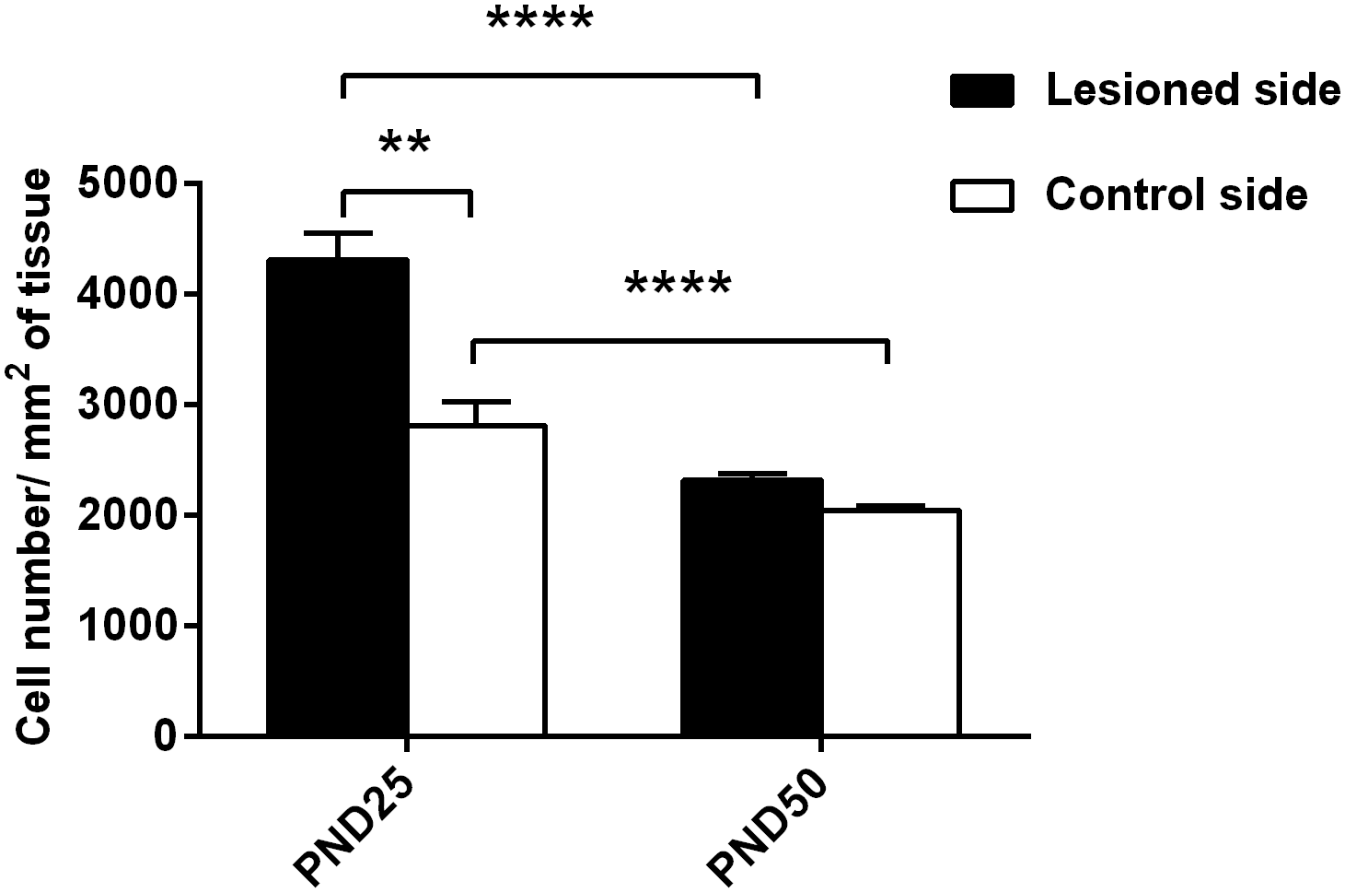
Total cell density in the lesioned area at PND25 and PND50. The total cell density in the area that had been interested by the lesion was calculated as number of nuclear staining-positive cells/ um^**2**^ of tissue in 18 samples taken from rats sacrificed after the lesion at PND25 or at PND50 after phenotype validation. Significant difference is indicated as **p<0.01, ****p<0.001.

The injection of 6-OHDA in the DS, performed at PND21, left an edema that is visible at PND25. At this timepoint, an increase in cell number in the lesioned side compared to control side is observed (mean diff. 1496, p<0.0001), but a reduction of DrD1 (mean difference 23.88, p<0.0001) and DRD2 positive cells (mean diff 22.59, p<0.0001) is observed in the area adjacent to the injection site compared to an analogous area of the control side (Figs 8 and 9). At PND50 there is no visible trace of the lesioned area (Figs 6 and 7), but there is a significant lower number of DrD1 in the lesioned side compared to control side (mean diff. 9.95, p<0.05) (Fig 8), that is not due to a lower cell number in the lesioned side (Fig 9) while no difference in DrD2 is encountered (Fig 8).

**Fig 10:**
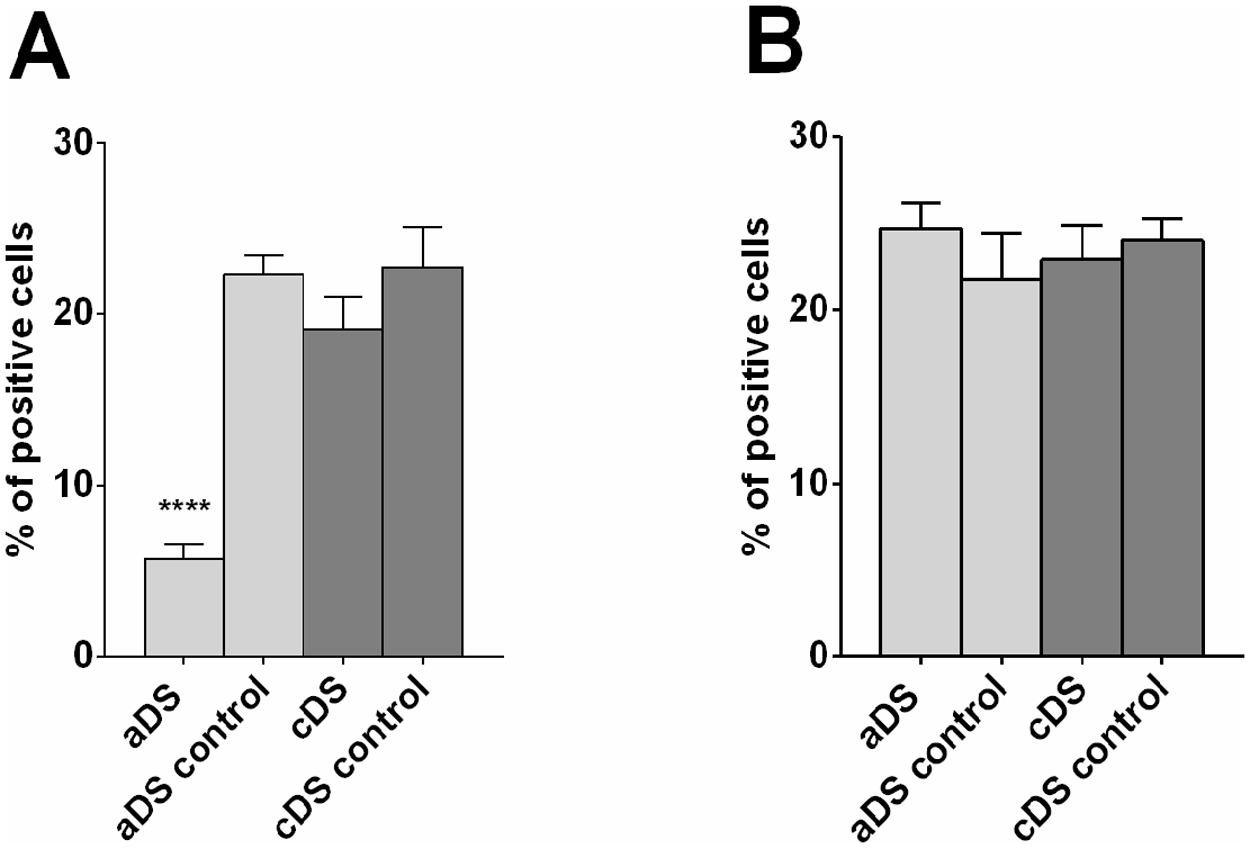
Preliminary analysis of DrD1 and DrD2 RNA positive cells in aDS and cDS of rats sacrificed at PND50. Cells positive for DrD1 RNA and nuclear staining (A) or DrD2 RNA and nuclear staining (B) were quantified in lesioned versus control side in aDS (n = 2) or cDS (n = 3) of DS lesioned rats and are reported as percentage of positive cells compared to total cells. Significant difference is indicated as **** p<0.001.

Preliminary results obtained with samples from aDS (n = 3) and cDS (n = 3) seem to indicate that DrD1 reduction in DS of PND50 rats is significant only in its anterior part (F_(3,8)_ = 23.64, p = 0.0002) (Fig 10).

### Glutamatergic modulation with riluzole effectively reduces tic-like phenotype

Glutamatergic corticostriatal projections are major activators of MSN activity and their activity is tightly interconnected with this of dopaminergic nigrostriatal neurons. Their possible role in the pathophysiology of tics and TS has been supported by clinical and preclinical data and anti-glutamatergic therapy has been suggested but remains poorly investigated [38–41].

We decided to investigate the effect of riluzole, a glutamatergic modulator, on DS-lesioned rats chronically administered with quinpirole.

**Fig 11:**
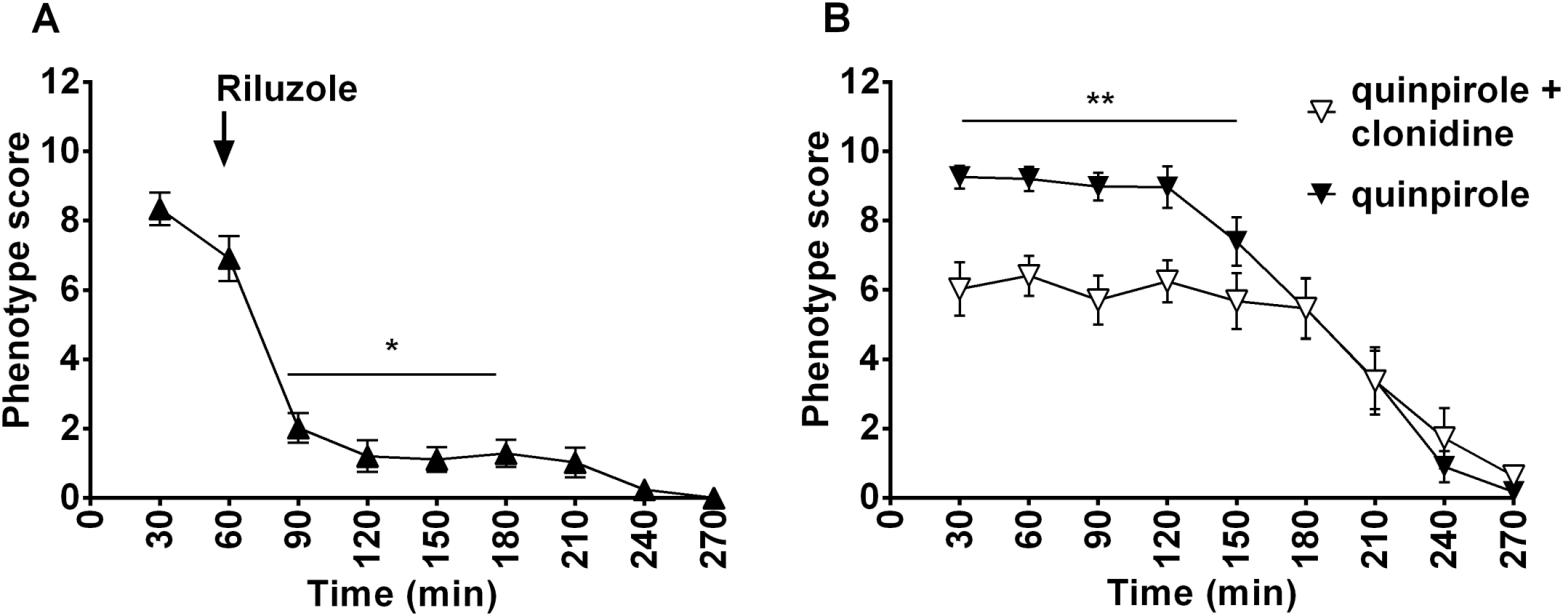
Time course of tic-like movements score after clonidine and riluzole administration. Clonidine (0.05 mg/kg IP) was administered together with quinpirole (A). Riluzole (6mg/kg IP) was administered 60min after quinpirole (indicated by an arrow) (B). Significant reduction of phenotype score compared to the score obtained during quinpirole treatment by the same animals is indicated as *p<0.05, **p<0.01.

At first, predictive validity was checked with clonidine an α2-adrenergic receptor agonist that is commonly used as a therapy for TS. A preliminary experiment with a higher dose of clonidine (0.5 mg/kg) was executed, but the high reduction of phenotype score observed appeared to be associated with signs of catalepsy, like rigidity and immobility (experimental observations) (S3 Fig). At a lower dose of clonidine (0.05 mg/kg), phenotype score was significantly different across treatment groups (F_(1,198)_ = 19.76, p<0.0001) and time points (F_(8,198)_ = 37.98, p<0.0001) with a significant interaction between treatment and time (F_(8,198)_ = 8.8, p = 0.0016) (Fig 11). In this case we did not observe sign of catalepsy.

Glutamatergic modulation with riluzole (6 mg/kg), administered 60 minutes after quinpirole due to its different pharmacokinetics, showed a rapid, significant reduction in tic-like movements score (F_(1,26)_ = 86.58, p<) 0.0001and no cataleptic effects (Fig 11 and S6 Fig)

## Discussion

This work aimed at understanding the role of dopaminergic alterations in the juvenile striatum, a brain region which is causally related to behavioral deficits of neurodevelopmental disorder Tourette’s syndrome (TS) [10,11,38]. In particular, we focused our attention on the dorsal region of the striatum which takes part in different aspects of motor function, including planning, learning and execution [37]. Alterations in this region have been previously associated with the generation of motor tics [16,30,31]. Consistent with literature findings [12,42], vocal tics, the other defining feature of TS, do not seem to be related to dorsal striatum abnormalities (S2 Fig).

Physiological movements are considered the result of a balanced integration of all components of the CSTC circuit, with particular relevance of the striatum as primary regulator of the circuit. A failure of physiological processes for selection of wanted movements to promote their execution could cause movement abnormalities [24,26,37].

The dopaminergic nigrostriatal system regulates movement through a continuous and strict balance of the striatal direct and indirect pathway dichotomy [24,26]. For these reasons, we introduced a dopaminergic imbalance in juvenile rats at the dorsal striatal level via lesion of the nigrostriatal terminals (S4 and S5 Figs) followed by a chronic administration of DrD2 agonist quinpirole. The protocol was developed to cover the phase of neurodevelopmental plasticity during the prepubertal phase in rats, in order to mimic the human condition [1,3].

Shortly after 6-OHDA injection in the DS, at PND25, the lesioned area appears characterized by tissue damage probably resulting from edema [43,44] and by increased cell number that likely reflects ongoing inflammatory processes [45]. Levels of both DrD1 and DrD2 mRNA expressing cells were markedly reduced (Figs 6 and 7), however, it is worth mentioning that no behavioral abnormality was observed at this time point. Movement alterations were first associated with the beginning of quinpirole chronic treatment, and became consistent after 6 quinpirole administrations on alternate days. The majority of movements appeared to be involuntary, fast, and patterned, similarly to tics in patients and we defined them tic-like movements (Table 1).

It is important to underline that the presence of face validity in rodent models of tics mostly rely on their adherence to the definition of tics established for humans (sudden, rapid, recurrent, nonrhythmic motor movements or vocalizations). The consideration of fast, repetitive and recurring movements as tic-like movements is a generally accepted compromise considering the fact that the identifying feature of tics in TS- waxing and waning course and their anticipation by sensory phenomena- are hard to be translated into preclinical models. However, as a consequence, different types of movements may fall under this definition and a careful observation is needed for their characterization and distinction. For instance, in patients, tics can be confounded with stereotypies, which are characterized by the execution of extremely rigid sequences of involuntary movements. In animal models, a well-described stereotype is the increase in chained grooming [31,46], that is now considered a rodent expression of compulsive behavior and was not observed in our animal model.

While the major components of the CSTC are conserved, the great differences in the movement repertoire of humans and rodents are likely to lead to a different manifestation of tics [47,48].

For this reason, we first carefully categorized abnormal movement manifestations and described those similar to tics, (Table 1) that represented the major phenotype.

Tic-like movements were shown to involve the paw and the mouth independently or form complex patterned movements also involving the neck (S1 Video) reflecting the distinction between simple and complex tics in patients. Other motor phenotypes like mild axial torsion and contralateral rotations were occasionally observed in DS and cDS lesioned rats, but did not represent a major phenotype. Contralateral rotation is considered as an effect of the intra-hemispheric difference of dopaminergic input introduced with the lesion [49]. Axial dystonia is a typical component of the dyskinetic phenotype of adult rats lesioned with 6-OHDA in the medial forebrain bundle. Our model shows very low signs of axial torsion, probably because of the relatively small lesion area. As the coexistence of TS and dystonia has been reported in the literature [50,51], DS or cDS lesioned rats could be a useful tool in the investigation of this condition.

As a rating scale for tic-like movements in animal models was missing but needed [52], we developed a new one by translating the one in use for patients.

The new rating system is focusing on movements with tic-like features in order to increase its translatable potential, while minor phenotypes were indirectly taken into account as impairing factors (Table 2). The distinction between simple and complex tic-like movements represents an innovative point of our rating system.

In our characterization we moved forward to determine the role of the anterior and the central DS in phenotype formation, knowing that the dorsal striatum is topographically organized [16,17,37]. We observed that aDS lesioned juvenile rats exclusively show mild simple tic-like movements of the mouth and the paw, while cDS lesioned juvenile rats have a more complex and marked phenotype, similarly to the full DS lesioned juvenile rats involving simple and complex movements of the paw, mouth and neck (Fig 5). This result may indicate that both DS regions are equally implicated in mouth movements, while mainly cDS is involved in neck and limb movements.

Bronfeld and colleagues showed tic-like movements evoked by microinjection of GABA antagonist bicuculline in the DS [16]. In their observations, mild facial movements were reported after both injections in the anterior and in the dorsal striatum, consistent with our findings. The major phenotypes they described were strong tic-like movements of forelimbs and hindlimbs depending on whether the area of injection was anterior dorsal striatum or posterior dorsal striatum respectively. We did not investigate the posterior dorsal striatum and did not observe hindlimb tic-like movements, but we reported forelimb tic-like movements associated with aDS as well, and cDS. Bicuculline is a GABA antagonist which is considered to relieve striatal MSN near the injection site from interneuronal inhibition. Consequently, striatal projection neurons become more susceptible to abnormal firing when stimulated by the premotor cortex. Our model acts at a different level of the CSTC circuit: by removing the control held by nigrostriatal DA projections on cortico-striatal synapses. We reckon that in both cases MSN are more easily subjected to abnormal firing, possibly causing tic-like movements.

In *post mortem* samples obtained from DS lesioned juvenile rats at PND50, the absence of DA afferent fibers to DS indicated the successful ablation of nigrostriatal neurons. At the striatal level, recovery of both DrD1 and DrD2 mRNA expression between PND25 and PND50 can be observed, although at a different extent: while the expression level of DrD2 mRNA at PND50 is comparable between lesioned and unlesioned side, DrD1 mRNA expression on the lesioned side is still significantly lower than on its control side (Fig 9). The majority of the studies investigating the expression level of DrD1 and DrD2 receptors at a late timepoint after 6-OHDA was mainly done in adult rats [23,53–56]. Fewer studies analyzed DRD1 and DRD2 expression in adult rats that received 6-OHDA as neonates [57–60] showing that the precise age at which the lesion was performed and the time between lesion and testing determine the experimental outcome. Our investigations covered a critical period for striatal development that takes place between PND20 and PND60, during which the number of DrD1 and DrD2 positive neurons is subjected to a continuous variation [60–63]. It is known that 6-OHDA lesions and receptor stimulation can interfere with the physiological development of DA receptors in the striatum [54,60,64]. For this reason, we think that the continuous agonism of DrD2 trough quinpirole could have supported DrD2 but not DrD1 expression, that remained lower in the lesioned side. The resulting imbalanced activation of direct and indirect pathway could represent the basis of the motor phenotype observed. In support of our results, DrD1 mRNA levels were reported to be reduced in TS patients by one of the few available *post mortem* investigations [65].

Furthermore, several studies suggested DRD2 as a candidate gene for TS [66–68], while imaging and *post mortem* studies supported abnormality of DrD2 expression in the striatum [69] or in other brain areas [70,71]. However, it needs to be mentioned that other studies failed to replicate these results [72,73] and the involvement of DrD2 in TS is still under debate.

On the other hand, it is clear that therapeutics targeting DrD2 effectively alleviate tics Currently they represent one of the few FDA-approved treatments for TS, although they are not the drug of choice of physicians because of their strong side effects [29,74].

We decided to address the role of glutamatergic regulation as possible strategy for tic reduction after increasing lines of evidence showed the involvement of the glutamatergic system in TS [39,41,75]. The glutamatergic modulator riluzole was shown to have a safe profile and was investigated in numerous child-onset psychiatric disorders with successful outcome [76–79]. In TS patients a single preliminary study showed tic reduction associated at a comparable extent to riluzole and placebo treatment. Because of intrinsic limitations of the study such as low number of participants and limited time-duration, the authors strongly recommend further investigations [79]. Riluzole has been successfully used as off label therapy for tic reduction, and for this reason we decided to support its investigation in our tic-like movements model.

Predictive validity was first tested in the presence of the alpha-adrenergic agonist clonidine, a therapeutic option for tics and comorbid attention deficit/ hyperactivity disorder in patients [80,81] that effectively reduced tic-like phenotype in quinpirole-treated DS-lesioned rats (Fig 3).

Upon riluzole treatment we observed a strong reduction of tic-like movements (Fig 11) that could result from a partial restoration of the delicate balance between dopamine and glutamate at the cortico-striatal projections level or to a compensatory action at the thalamo-cortical level. In our observations, animals were able to move freely and showed no sign of catalepsy thus suggesting that glutamatergic modulation could represent a safe therapeutic option for TS patients.

## Supporting information

S1 Fig Food intake of DS lesioned rats administered with quinpirole and saline. (TIF)

S2 Fig Ultrasonic vocalizations produced by rats treated with quinpirole. USV produced by DS (n = 15), aDS (n = 15), cDS (n = 15) and sham (n = 15) lesioned rats administered with quinpirole are characterized according to their number (A) duration (B) average frequency (C) amplitude (D) and call types expressed in percentage of the total number (E). Significant difference between groups is indicated as *p<0.05.
(TIF)

S3 Fig Time course of tic-like phenotype of quinpirole treaded DS lesioned rats administered with 0.5 mg/kg of clonidine. (TIF)

S4 Fig Lesion of the anterior DS. The lesioned area could be observed through tyrosine hydroxylase (TH) immunostaining, revealing loss of dopaminergic TH positive projections in 6-OHDA lesioned aDS.
(TIF)

S5 Fig Lesion of the cDS. The lesioned area could be observed through tyrosine hydroxylase (TH) immunostaining, revealing loss of dopaminergic TH positive projections in 6-OHDA lesioned cDS.
(JPG)

S6 Fig Catalepsy is not associated to riluzole treatment in rats. DS lesioned rats were administered with saline, HA (0.1, 0.5 and 1 mg/kg) and riluzole (6 mg/kg) 45 min after quinpirole administration. Time of catalepsy was calculated as time the animals spent on a horizontal bar without movement, for a maximum of one minute. Significant difference between groups is indicated as: *p<0.05, **p<0.01, ***p<0.005 and ****p<0.01.
(TIF)

S1 Video Example of simple tic-like movements. (AVI)
