# Supplementary figures and images for: Altered dopaminergic regulation of the dorsal striatum is able to induce tic-like movements in juvenile rats
Source: PLoS One. 2018 Apr 26;13(4):e0196515. doi: 10.1371/journal.pone.0196515 (PMC5919623; doi:10.1371/journal.pone.0196515)

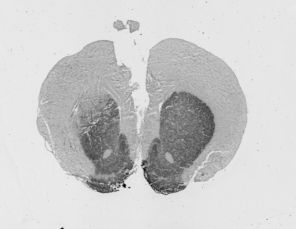

Supplement: S4 Fig — The lesioned area could be observed through tyrosine hydroxylase (TH) immunostaining, revealing loss of dopaminergic TH positive projections in 6-OHDA lesioned aDS. (TIF) [file pone.0196515.s004.tif]

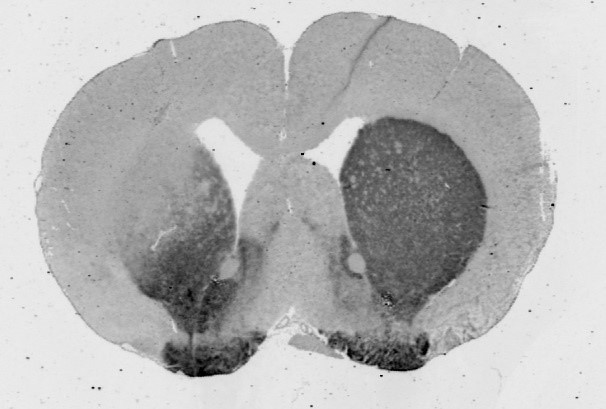

Supplement: S5 Fig — The lesioned area could be observed through tyrosine hydroxylase (TH) immunostaining, revealing loss of dopaminergic TH positive projections in 6-OHDA lesioned cDS. (JPG) [file pone.0196515.s005.jpg]
